# Strategies for disseminating recommendations or guidelines to patients: a systematic review

**DOI:** 10.1186/s13012-016-0447-x

**Published:** 2016-06-07

**Authors:** K. Schipper, M. Bakker, M. De Wit, J. C. F. Ket, T. A. Abma

**Affiliations:** 1 Department of Medical Humanities, VU University Medical Center, Amsterdam, The Netherlands; 2 EULAR Standing Committee of People with Arthritis/Rheumatism in Europe, Zurich, Switzerland; 3 Medical Library, VU University, Amsterdam, The Netherlands; 4 Department of Medical Humanities/EMGO+, VU University Medical Center, Post box 7057, 1081 BT Amsterdam, The Netherlands

**Keywords:** Systematic literature review, Dissemination, Guideline(s), Recommendation(s), Patient(s), Patient organisation(s), Involvement

## Abstract

**Background:**

The aim of this systematic literature review was to assess what dissemination strategies are feasible to inform and educate patients about recommendations (also known as guidelines).

**Methods:**

The search was performed in February 2016 in PubMed, Ebsco/PsycINFO, Ebsco/CINAHL and Embase. Studies evaluating dissemination strategies, involving patients and/or reaching patients, were included. A hand search and a search in the grey literature, also done in February 2016, were added. Searches were not restricted by language or publication type.

Publications that referred to (1) guideline(s) or recommendation(s), (2) dissemination, (3) dissemination with patients/patient organisations and (4) dissemination to patients/patient organisations were included in this article. Criteria 1 AND 2 were mandatory together with criteria 3 OR 4.

**Results:**

The initial search revealed 3753 unique publications. Forty-seven articles met the inclusion criteria and were selected for detailed review. The hand search and grey literature resulted in four relevant articles. After reading the full text of the 47 articles, 21 were relevant for answering our research question. Most publications had low levels of evidence, 3 or 4 of the Oxford levels of evidence. One article had a level of evidence of 2(b).

This article gives an overview of tools and strategies to disseminate recommendations to patients. Key factors of success were a dissemination plan, written at the start of the recommendation development process, involvement of patients in this development process and the use of a combination of traditional and innovative dissemination tools. The lack of strong evidence calls for more research of the effectiveness of different dissemination strategies as well as the barriers for implementing a strategic approach of dissemination.

**Conclusion:**

Our findings provide the first systematic overview of tools and strategies to disseminate recommendations to patients and patient organisations. Participation of patients in the whole process is one of the most important findings. These findings are relevant to develop, implement and evaluate more (effective) dissemination strategies which can improve health care.

**Electronic supplementary material:**

The online version of this article (doi:10.1186/s13012-016-0447-x) contains supplementary material, which is available to authorized users.

## Background

In health care, many guidelines or recommendations for the management of diseases are developed. These recommendations are primarily developed to inform health professionals to improve daily routines of medicine. Dissemination and implementation of these recommendations are often focussed on professionals [1], not on patients. As a result, in many countries, patients are not aware of the existence of recommendations, are not able to access the publications or do not fully understand the English language and the academic and medical terminology. Patients have therefore limited access to information to get an adequate understanding of their disease and treatment options. One way to empower patients to make more informed choices is the development and dissemination of patient or lay versions of the recommendations. Providing lay versions might be seen as a key component of good care [2], especially because patients increasingly want to be involved in decision-making processes [3]. Involving good-informed patients in their treatment decisions is assumed to lead to more personal comfort with the treatment decision [2], better treatment adherence and motivation, reduction of the number of interventions in some cases [4] and more control by patients [5].

Improvement of health care can be enhanced by the dissemination of recommendations that are easy to find and easy to understand by patients. Making these recommendations accessible for patients requires an extra effort from health professionals or patient organisations to translate the English version into another language and to adjust the content of the recommendations to the national context, and the specific information needs of patients without losing scientific rigor [6].

Studies on the dissemination of recommendations towards professionals are extensively described in the literature (e.g. [7–12]). However, it is not systematically investigated which strategies are feasible for the dissemination of recommendations to patients. The aim of this systematic literature review is to assess the feasibility of dissemination strategies to inform and educate patients about recommendations or guidelines. This review is part of the European League Against Rheumatism (EULAR) project to develop a practical guide for patient organisations to improve the dissemination of EULAR recommendations to people with rheumatic and musculoskeletal diseases. This review will hopefully enable other national organisations of patients and health professionals to develop their own strategy to disseminate national or international recommendations to patients. In the context of this review, the word guidelines and recommendations are used as synonyms.

### Research question

What dissemination strategies are feasible to inform and educate patients about recommendations or guidelines?

## Methods

### Searches

This systematic literature review (SLR) followed the process recommended by the Centre of Reviews and Dissemination [13]. The scope of the SLR was discussed by a EULAR Task Force representing eight countries, covering all regions of Europe. It comprised seven patient experts, six health professionals (three rheumatologists, three health professionals) and one dissemination expert. The group followed the EULAR Standardized Operational Procedures [14] and met twice.

The search terms and strategies were discussed in the research team (TA, MB, KS, MdW). A review protocol was developed by KS and JK, based on the Preferred Reporting Items for Systematic Reviews and Meta-Analysis (PRISMA) statement [15]. PubMed, Ebsco/PsycInfo, Embase.com and Ebsco/Cinahl were searched on 4 February 2016, all from inception, by KS and JK. The following terms were used (including synonyms and closely related words) as index terms or free-text words: ‘guidelines’ or ‘recommendations’ and ‘dissemination’ and ‘patients’ or ‘consumers’. The full search strategies for all databases can be found in Additional file 1. Searches were not restricted by language, publication type or date.

Duplicate articles were excluded. All languages were accepted. A search in the grey literature was added, using the method of ‘communication with experts’ and ‘snowballing’ [16, 17]. The reference lists of articles from the search that fit the criteria were scanned for missing papers.

### Study inclusion criteria

To be included in our final article, the article had to refer to (1) guideline(s) or recommendation(s), (2) dissemination, (3) dissemination with patients/patient organisations and (4) dissemination for patients/patient organisations. Criteria 1 AND 2 were mandatory together with criteria 3 OR 4.

### Study selection and data extraction

After deleting duplicates, all articles (title and abstract) were screened for inclusion, independently by two reviewers (KS and MB). Discrepancies were resolved by discussion, supported by two of the authors (TA and MdW). Abstracts that met the inclusion criteria were selected for detailed, full text review. Reasons for exclusion were dissemination towards professionals instead of patients/patient organisations. The selected abstracts were complemented by articles and grey literature identified through a hand search.

### Data synthesis and presentation

We found qualitative studies, surveys, descriptive studies, opinions, editorials and conference abstracts. Because of the nature of these studies, a statistical synthesis was not appropriate. Therefore, a content analysis [18] was done and themes were extracted, using codes. Both reviewers (KS and MB) conducted the analysis separately and then explored similarities and differences between the studies. The research team then synthesised and interpreted the evidence as it related to the purpose and aims of the review.

### Quality assessment

Two reviewers independently assessed the methodological quality of included studies (KS, MB). The Quality Assessment Tool for Quantitative Studies was used for assessing the quality of the quantitative studies [17] (see Additional file 2). This tool leads to an overall methodological rating (strong, moderate or weak) taking important elements into account such as selection bias, study design, confounders, blinding, data collection methods, withdrawals/dropouts, intervention integrity and analysis [17].

We used the Quality Assessment Tool for Qualitative Studies to assess the methodological quality of the qualitative studies [19] (see Additional file 3). Five aspects were taken into account: the aims of the research; research methods and design; sampling; data collection and analysis and results, discussion and conclusions. This tool has similarities with the tool for assessing quantitative research and is recommended by Cochrane.

The level of evidence was categorised according to the design characteristics of available studies using an established hierarchy [20] (see Table 1).

**Table 1:** Categories of evidence [20]

Level of evidence	Study
1A	From meta-analysis of randomised controlled trials
1B	From at least one randomised controlled trial
2A	From at least one controlled study without randomisation
2B	From at least one type of quasi-experimental study
3	From descriptive studies, such as comparative studies, correlation studies or case-control studies
4	From expert committee reports or opinions and/or clinical experiences of respected authorities

## Results

**Fig. 1:**
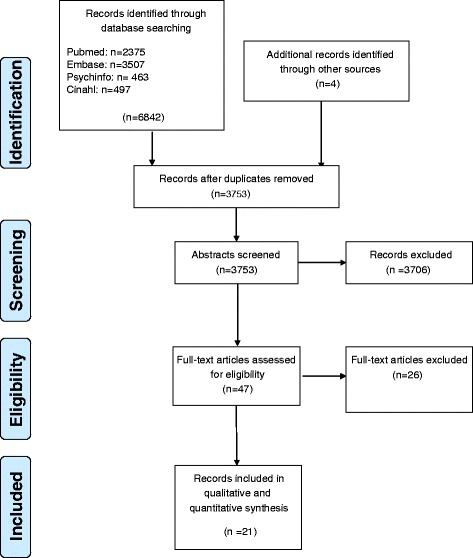
Flowchart

In total, we identified 47 articles that met the inclusion criteria, 43 through the SLR and 4 through the hand search. After reading the full text, 21 articles were included in this review (see Fig. 1). The articles are published between 2002 and 2014 but most of them are published between 2010 and 2014 (15). Most of the articles are published in 2013 (8 articles). The articles are mainly about the field of rheumatology, asthma/COPD and diabetes. The authors are from the Canada (7), Europe (7), USA (5), Russia (1) and Africa (1).

Most of the 21 papers can be described as opinion papers or papers that describe strategies that have been used or might be effective. Only a small amount of papers are based on a RCT or a survey or qualitative study. The 21 papers were assessed for the level of evidence and were scored level 3 (descriptive studies, such as comparative studies, correlation studies or case-control studies) or 4 (expert committee reports or opinions; Table 2). Only one study was assessed as having a level of evidence of 2B (controlled study). Although Cochrane suggests to exclude descriptive papers, editorials or opinion papers, we have included those papers because of the lack of articles with a level of evidence of 1 (meta-analysis of randomised controlled trials) or 2 (single randomised controlled trials). The analysis of the articles showed that, besides information about dissemination to patients or patient organisations, many articles paid attention to patient involvement in dissemination. This result section will, therefore, address both themes: information on dissemination to patients or patient organisations and patient involvement in dissemination.

**Table 2:** Characteristics of selected papers

	Authors	Title	Journal	Kind of article	Level of evidence	Dissemination strategy as evaluated in article and/or results
1	Abrahamian et al.	Strategies for health system implementation of guidelines on overweight and obesity.	BMJ Qual Saf. 2013; 22(Suppl 1): A34-A34.	Description of used strategies.	3/4	Patient-level interventions like proactive outreach for health education classes and telephone-based coaching, point-of-care educational publications, and after-visit summaries with weight management recommendations.
						Continued improvements in clinician/patient communication about weight, collection of patient weight information and patient health outcomes have been observed.
2	Allu et al.	Hypertension: Are you and your patients up to date?	Can J Cardiol 2010;26(5): 261-264.	Opinions of authors based on their experiences. Their opinions are furthermore based on literature about knowledge translation research.	4	CHEP (Canadian Hypertension Education Program) and BPC (Blood Pressure Canada) are developing a series of innovative new programmes to try to enhance hypertension knowledge translation and dissemination. It consists of a combination of methods: website, email notices, interactive internet-based lectures, community hypertension champions, patient association and variety of learning tools (posters, summaries, handouts, pocket cards, standardised slide sets).
3	Appiah et al.	Challenges and opportunities for implementing diabetes self-management guidelines.	JABFM January–February 2013 Vol. 26 No. 1 90-92.	Qualitative study consisting of 3 group discussions of professionals.	3	Key themes that emerged as challenges of implementing evidence-based guidelines included lack of easily retrievable electronic patient health information, inadequate coordination with other health care providers when implementing guidelines, conflict between information in the guidelines and physicians’ knowledge and physician compensation by patient load rather than by quality of care.
						Opportunities that were mentioned: the use of health coaches or nurses trained in diabetes self-management and active collaboration between practicing providers and key stakeholders in the development and dissemination of guidelines.
4	Azevedo et al.	Control of Allergic Rhinitis and Asthma Test (CARAT): dissemination and applications in primary care.	Prim Care Respir. 2013; 22(1): 112-116.	Description of strategies and opinion based on international recommendations and best practices.	3/4	Widespread availability of cross-cultural adaptations, print, web and mobile versions, a free open model of distribution, user support through a dedicated website, clinical educational sessions and dialogue with health authorities and integration into clinical guidelines.
5	Boulet et al.	Implementing practice guidelines: a workshop on guideline dissemination and implementation with a focus on asthma and COPD.	Can Respir J 2006;13(Suppl A):5A-47A.	Opinions of authors, based on a workshop with leading professionals.	4	Potential successful strategies according to leading professionals are:
						Making a dissemination plan in parallel with the development of the recommendations.
						Making a lay version that is customised to the target audience.
						Making the lay version relevant for the target audience
						Making consistent, unambiguous and credible lay versions
						Making lay versions that provide clear, explicit and specific information.
						Making lay versions that contain information about where more in-depth information can be found.
						Using a combination of dissemination strategies like : organising press conferences, providing lay versions through Public Libraries, developing books to reach children and developing posters with ‘trigger’ stickers or making a web site endorsed by a VIP.
6	Drouin et al.	Dissemination and implementation of recommendations on hypertension: the Canadian experience.	Allergy, Asthma & Clinical Immunology 2010, 6 (Suppl 4):A10.	Description of experiences/ideas/opinions of authors.	4	Dissemination requires a combination of different, mutually reinforcing strategies.
7	Eccles et al.	Developing clinical practice guidelines: target audiences, identifying topics for guidelines, guideline group composition and functioning and conflicts of interest.	Implementation Science 2012, 7:60.	Opinions mainly based on a Systematic Literature Search of Legare and colleagues (2011).	3/4	Dissemination is more successful if patients are involved in the development of guidelines.
						The involvement of patients increases the comprehensiveness of the recommendations and makes the adaption of the recommendations to the target population easier.
						It is important to use selection criteria in choosing patient representatives.
						Patients can also be involved in less traditional ways.
						Choose for more innovative ways such as the use of new media that better fit the patients’ role, expectations and capabilities.
						Involve a heterogeneous group of patients in order to communicate to a diversity of patients.
						Training and support can be helpful to make the involvement of patients in the development and dissemination of recommendations successful.
8	Eijk van et al.	Dissemination and evaluation of the EULAR recommendations for the role of the nurse in the management of chronic inflammatory arthritis. Results of a multinational survey among nurses rheumatologists and patients.	Rheumatology 2014; 53(8): 1491-1496.	Description of results of a multinational survey among nurses rheumatologists and patients about the dissemination of recommendations.	3	An internet survey was used in order to disseminate recommendation among nurses, rheumatologists and patients. Snowball sampling was used to reach as much people as possible.
						A successful change of clinical practice in accordance with the recommendations requires an effective implementation strategy in which the key stakeholders delivering and receiving care (among others patients), are involved in the dissemination.
9	Gainforth et al.	Examining the effectiveness of a knowledge mobilisation. initiative for disseminating the physical activity guidelines for people with spinal cord injury.	Disabil. Health. 2013; 6 (3):260-265.	Description of results of a questionnaire aimed at the examination of the reach and effectiveness of an event-based KM initiative .	3	An event-based KM initiative may be effective for initial dissemination of guidelines. Efforts are needed to foster long-term guideline adoption.
10	G-I-N-Public working group.	G-I-N Public Toolkit. Patient and public involvement in guidelines.	-	Toolkit consisting of information about patient involvement in guidelines. The information based on a series of consultation activities held by the working group at international conferences of the Guidelines International Network. The knowledge generated by the consultation, the work and the experience of the members of the working group and literature on the topic formed the basis for developing the toolkit.	4	Lay versions of guidelines should be made by patient organisations, using a heterogeneous group of patients with different disease status and educational levels.
						Patients who have participated in the development of the recommendations can also actively contribute to the dissemination process
						The establishment of permanent groups, networks or ‘virtual panels’ of patients can help to disseminate guidelines. The network members are alerted when new recommendations or patient versions are published. They can raise awareness by distributing lay versions to health professionals, patients, patient organisations and members of the public.
						Patients involved in the dissemination process have specific needs that should be taken into account. Training and support is an important need.
						Lay versions should be disseminated by National patient organisations by using their own website, newsletters, brochures, other publications, phone calls, support groups, workshops, events, seminars, annual conferences, local or regional events, events for professionals and/or patients, press releases, print-ready ads, fillers or by including the recommendations in their information packages provided to their members.
						Personal stories of patients in media can help to raise awareness of new recommendations
11	Guyatt et al.	GRADE guidelines: a new series of articles in the Journal of Clinical Epidemiology.	Journal of clinical epidemiology, 2011. 64(4): p. 380-2.	The “Grades of Recommendation, Assessment, Development, and Evaluation” (GRADE) approach provides guidance for rating quality of evidence and grading strength of recommendations in health care.	4	Guidelines developed for resource-rich countries are often inapplicable in resource-poor countries.
12	Hoens et al.	Knowledge brokering: an innovative model for supporting evidence-informed practice in respiratory care.	Can.Respir.J. 2013; 20 (4): 271-274.	Description of the role of a KB in health care.	4	The use of knowledge brokers (KB) which are individuals who work to bridge the gap between researchers and knowledge users. In the health care setting, KBs work closely with clinicians to facilitate enhanced uptake of research findings into clinical practice. They also work with researchers to ensure research findings are translatable and meaningful to clinical practice.
						The KB role has provided an important communication link between researcher and knowledge user that has facilitated evidence-informed practice to improve patient care.
13	Jae Jeong et al.	Major cultural-compatibility complex: considerations on cross-cultural dissemination of patient safety programmes.	BMJ Qual Saf 2012 21: 612-615.	Description of experiences/ideas/opinions of authors based on own experiences .	4	Cultural differences should be taken into account when disseminating guidelines to other countries.
						Careful consideration should be given to social and cultural sensitivities and differences. Success in one country does not guarantee success in other countries.
						The first step in any effort to reach a new public is to thoroughly understand the culture and cultural diversity of the target audience.
						Adaptation of the recommendations to the local situation may be needed.
14	Ke et al.	Disseminating the Canadian diabetes association 2013 clinical practice guidelines: Guidelines. Diabetes.ca in action.	Can J Diabetes. 2014; 38: S72-S73.	Description of strategies.	4	An electronic point-of-care tools, templates, laboratory prompts and a communications campaign, complemented by minimal hardcopy resources. Electronic tools (available at guidelines.diabetes.ca) include easily-searchable guidelines with slide set summaries and video narrations, brief reference guides, interactive decision-support algorithms, flow sheets and patient self-management tool.
						Results: In a 6-month period, there have been 190 291 views from around the world, with the average user spending up to 5 min on the site. For guidelines to have an impact on patients, they must be effectively integrated into clinical care. In this digital era, this necessitates electronic point-of-care tools, usable and immediately accessible information resources, and a recognised web presence.
15	Kiltz et al.	ASAS/EULAR recommendations for the management of ankylosing spondylitis: the patient version.	Ann Rheum Dis. 2009 Sep;68(9):1381-6.	Description of experiences with making a lay version of recommendation together with patients.	3/4	In cooperation with patient organisations, 18 patients were invited to attend a meeting. As a starting point the original publication and a version created by Canadian patients was used. After intensive discussions, the wording was adjusted and a vote was held on the new wording of the recommendations aiming for >80 % agreement on each sentence. Finally, patients were asked to indicate their level of agreement with the content of the recommendations
						Ten recommendations were successfully translated into a patient-understandable version. The original text was changed in most cases. In all but one case, there was broad agreement with the proposed translation. The overall agreement with the content of the recommendations was high.
16	Maximov et al.	Implementation of the osteoarthritis clinical guideline: Results of a cluster randomised trial in primary care.	Ann Rheum Dis. 2013; 71:307-308.	RCT of dissemination of guideline.	2	One-day didactic educational meeting, provision of the printed guideline and patient brochures.
						The implementation of the clinical guideline by means of the didactic educational meeting in combination with dissemination of the printed guideline and patient brochures may optimise treatment and improve patient outcomes in a long-term perspective, but trials with a greater sample size are needed to confirm this effect more precisely.
17	McGuire et al.	Promulgation of guidelines for mucositis management: educating health care professionals and patients.	Support Care Cancer. 2006: 14: 548–557.	Survey among cancer health care professionals on dissemination.	3	Awareness of the guidelines of professionals is limited in the US, and use of the guidelines worldwide is minimal.
						Information for patients is often too difficult for the general public. Information can be simplified by using less medical and technical terms or by giving an explanation of the terms.
						Patients do not speak the same language as health professionals.
						The use of familiar words of one or two syllables, the use of active voice in the present tense and the use of short sentences of 15 words or less, and short paragraphs of 10 lines or less may help to make information more readable
18	Sharpe et al.	Development of culturally tailored educational brochures on HPV and Pap tests for American Indian women.	J Transcult Nurs. 2013; 24 (3):282-290.	Opinion.	4	A participatory process successfully engaged nursing staff and patients in creating culturally appropriate brochures for clinic use.
19	Snyman.	Using the printed medium to disseminate information about psychiatric disorders.	South African Psychiatry Review.7(4) 15-20 2004.	Text-focused evaluation method, using the adapted version of the suitability assessment of material (SAM-test) to evaluate the effectiveness of brochures disseminating information to patients.	3	The findings indicate to which degree brochures about schizophrenia do not meet general accepted criteria for effective printed health messages. The readability level of the brochures indicated a target audience of at least university graduates which makes them unsuitable as information material for the general South African public.
20	Tulder van et al.	Disseminating and implementing the results of back pain research in primary care.	SPINE Volume 27, Number 5, pp E121–E127. 2002.	Opinions mainly based on a workshop with leading experts on the area of dissemination.	4	The involvement of patients increases the comprehensiveness of the recommendations and makes the adaption of the recommendations to the target population more easy.
						Lay versions should be developed in order to disseminate guidelines to patients.
						A successful lay version provides clear, explicit and specific information.
						Recommendations should be readable, comprehensible, relevant, consistent, unambiguous and credible to increase the success of the dissemination.
						Information about where more in-depth information can be found should be included in the lay version.
						The use of passive dissemination strategies, such as a leaflet or brochure is insufficient to educate patients or change daily routine because such information does not endure in the long term.
21	Vandvik et al.	Creating clinical practice guidelines we can trust, use, and share: a new era is imminent.	CHEST. 2013; 144 (2):381-389.	Opinion and description of experiences.	4	An online application that constitutes an authoring and publication platform that allows guideline content to be written and structured in a database, published directly and that includes electronic medical record systems, web portals, and applications for smartphones/tablets. This system allows automatic updates.
22	No author mentioned.	Involving patients and the public in implementing NICE guidance.	http://webarchive.nationalarchives.jsp	Suggestions on how to involve patients, based on own experiences.	4	The involvement of patients increases the comprehensiveness of the recommendations and makes the adaption of the recommendations to the target population easier.
						Ideally, a heterogeneous group of patients with different educational levels should be involved in order to communicate to a diversity of patients.

### Dissemination to patients or patient organisations

#### Dissemination plan

The search gives insight in three main factors that may make the dissemination of recommendations towards patients more successful. The first factor concerns the development of a dissemination plan [21, 22]. An adequate strategy requires, according to Boulet et al. [6] and Allu et al. [21], a dissemination plan that is ideally developed in parallel with the development of the recommendations; the plan should be made during the project and not at the end of the project [6, 21]. A dissemination plan is needed to clarify at the start of the project the target audience, which will subsequently determine the scope, objectives, format, style and wording of the recommendations as well as the tools for dissemination [22].

### Lay version

Producing a lay version of the original recommendations is the second factor that may improve the dissemination of recommendations [6]. A lay version enables patients to better understand the goals of treatment, the different treatment options and the benefits and risks of each option. Patients who have access to lay versions are better equipped to prepare themselves for the consultation with their health care provider and are expected to become an active partner in their own treatment [6].

Boulet et al. recommend to take the following aspects into account when developing a lay version. First, the message should be customised to the target audience. The information should be made relevant for the target audience, patients in this case. Furthermore, the information in the recommendations should be consistent, unambiguous and credible [6]. A successful lay version provides clear, explicit and specific information [6, 23] and some key messages [2]. The information in the lay version has to be readable for patients. A well-known pitfall is that information is often too difficult for the general public [24, 25] and in particular for less literate persons [25]. Information can be simplified by using less medical and technical terms or by giving an explanation of the terms [24]. McGuire et al. stress the fact that patients do not speak the same language as health professionals [24]. Only after a while, they will become more familiar with the language spoken by professionals [24]. Based on a survey among professionals, McGuire et al. further recommend the use of familiar words of one or two syllables, the use of active voice in the present tense and the use of short sentences of 15 words or less, and short paragraphs of ten lines or less [24]. Finally, information about where more in-depth information can be found should be included in the lay version [6, 23].

For international guidelines, lay versions of guidelines should ideally be translated into different languages. Based on international recommendations and best practices, Azevedo and colleagues [26] suggest to follow three steps for the translation and cross-cultural adaptation of guidelines: forward translation, back translation and patient testing. In the forward translation step, two professionals/patients (no translators) independently translate the original version into the target language. The translations are then compared, and an agreed version is drawn up between the translators and those involved in the development of the original version. In the back translation step, the text is translated back into the original language with the support of the developers of the original version. It is then compared with the original and reviewed to ensure conceptual equivalence. The last step is the patient test phase. Ten adult patients are given the translated version and are interviewed about the interpretation and wording of each item. The results are reviewed, and any changes are integrated into the third and final version [26].

### Combining strategies

The third factor that may lead to better dissemination is the simultaneous use of multiple tools and strategies. The use of different approaches can help to increase awareness and use among target populations [26].

Lay versions, as described above, are expected to reach individual patients. However, a study of Snyman suggests that just making a lay version is not enough to achieve this goal because most printed health messages do not transfer information successfully to target audiences [25]. The use of passive dissemination strategies, such as a leaflet or brochure, has proven to be insufficient to educate patients or change daily routine because such information does not endure in the long term [23]. Snyman’s study shows that strategies to disseminate lay versions need to be accompanied by the development of other materials. Several papers confirm that dissemination requires a combination of different, mutually reinforcing strategies (e.g. [6, 21, 26, 27]), for example, the repetition of key messages from different credible sources such as well-known professionals. Boulet et al. mention, based on their own experiences, the combination of the following strategies: organising press conferences, providing lay versions through Public Libraries, developing books to reach children and developing posters with ‘trigger’ stickers or making a website endorsed by a VIP [6].

Patient organisations can furthermore organise an annual national forum on a disease at which people share their experiences and take part in training and education programmes. Patient organisations can also provide telephone and online counselling and literature and other resources for patients and caregivers [6]. Allu et al. suggest to disseminate recommendations by providing automatic updates of new information and resources for patients who have signed in, by interactive internet-based lectures and by developing a variety of learning tools like posters, summaries, handouts, pocket cards and slide sets for patients [21]. Education to patients should furthermore be pro-active whereby face-to-face and contact by telephone can be used [28], just as education events for patients [29]. The use of internet and digital tools like websites and apps seems to be promising and necessary to reach patients [30, 31]. Guidelines should be easily searchable and accessible immediately [30, 31]. Another option is, according to Boulet et al., the development of community ‘champions’: through train-the-trainer sessions, community leaders are trained to become ‘champions’. Those champions assist in the dissemination of information to patients [6]. Knowledge brokers can also be used. Knowledge brokers are persons who bridge the gap between researchers and the end users [32]. Allu et al. stress the importance of patient associations. Patients should be encouraged to become a member of the association and receive information and other support [21].

When different sources and tools are applied over a longer period, the dissemination can be described as most adequate because information will last longer when seen and heard more often [6]. There is less information about the impact of the use of different strategies. The reviewed publications provide clear descriptions of the used or recommended dissemination strategies, but it is mostly unknown to what extent these strategies were effective. We do, however, know that event-based knowledge mobilisation may be effective for the initial dissemination of guidelines. For sustainable adaptation, more efforts are needed [29].

The literature also describes a study in which patient outcomes were improved by the dissemination of guidelines through didactic educational meetings, a printed guideline and a patient brochure [33], but we do not know which of these elements has resulted in the improvements.

To choose the right dissemination strategies means thus to combine passive and active strategies. According to Jeong et al., it is thereby necessary to take cultural differences into account [34] and to make versions bilingual in countries in which people speak different languages [20]. A lack of attention to cultural differences can lead to products or programmes that do not meet the needs or possibilities of the target audience. One of those issues is the risk that the programmes do not fit the recipient in terms of their unique culture. Success in one country does not guarantee success in other countries [34], or even within countries if there are significant cultural differences within countries. This is confirmed by the GRADE guidelines that emphasise the importance of the context. Guidelines developed for resource-rich countries are often inapplicable in resource-poor countries [35]. Here, careful consideration should be given to social and cultural sensitivities and differences like hierarchal culture, working according to plans or not [34], or the presence of certain professionals (for example specialised nurses) or health resources [34]. Therefore, the first step in any effort to reach a new public is to thoroughly understand the culture and cultural diversity of the target audience. Adaptation of the recommendations to the local situation may be needed [34].

In conclusion, in order to be as successful as possible, dissemination strategies should be characterised by developing a dissemination plan at an early stage, developing a lay version and using multiple dissemination strategies to reach the patients. The overriding principle to make recommendations accessible for patients and to ensure that they are comprehensible and fit the context is that the dissemination strategy suits the target audience. It is stressed by several authors that this can best be achieved by direct involvement of the target audience: patients and their organisations [22–25, 36]. How patients and their organisations can be involved is described below.

### Patient involvement

#### In developing recommendations

Several authors stress, based on their own experiences and ideas [1, 22–25] or a qualitative study among professionals [36], the importance of the involvement of patients or patient organisations in the design and development of recommendations to enhance the dissemination of recommendations in health care and to local patient organisations. Patients should be involved from an ethical point of view: involvement is needed to give patients influence on the recommendations by incorporating their experiential knowledge and perspectives. The involvement of patients, if done properly, increases the comprehensiveness of the recommendations because patients use other words and less jargon compared to professionals and their involvement makes the adaption of the recommendations to the target population easier because of their ‘patient knowledge’ [22, 23, 37].

An international working group of researchers, health professionals and patient representatives of the Guidelines International Network (GIN) has developed a toolkit about patient involvement in guidelines. The GIN supports patient involvement in guideline activities around the world. The toolkit is the result of a series of consultations, a literature review and the practice and experience of the GIN members [38].

#### In developing lay versions

There are different ways that patients or patient organisations can be involved in making a lay version [1]. EULAR has chosen to involve patients from different countries because of the international context of the recommendations. They have experienced that the involvement of patients with different native tongues enhances the likelihood that the lay version can be easily understood by many patients and that an English lay version can easily be translated into various languages because typical English phrasings are avoided. The GIN toolkit suggests that the translation of the English lay version in different languages should be done by patient organisations, using a heterogeneous group of patients with different disease status and educational levels [38]. Other authors [39] suggest to use a participatory (action) research (PAR) in order to involve patients in the development of guidelines/lay versions. Using such a PAR design may, according to the authors, result in culturally appropriate brochures for patients [39].

#### In disseminating recommendations

Van Eijk and colleagues state that a successful change of clinical practice in accordance with the recommendations requires an adequate or even better effective implementation strategy in which the key stakeholders delivering and receiving care (among others patients) are involved in the dissemination [40]. National patient organisations should, according to the GIN toolkit, disseminate the recommendations in their own countries. This can be done by using their own website, newsletters, brochures, other publications, phone calls, support groups, workshops, events, seminars, annual conferences, local or regional events, events for professionals and/or patients, press releases, print-ready ads, fillers or by including the recommendations in their information packages provided to their members [38]. Personal stories of patients in media can also help to raise awareness of new recommendations [38].

#### Who should be involved?

The GIN toolkit suggests that patients who have participated in the development of the recommendations can also actively contribute to the dissemination process [38]. Another suggestion is the establishment of permanent groups, networks or ‘virtual panels’ of patients [38]. The network members are alerted when new recommendations or patient versions are published. They can raise awareness by distributing lay versions to health professionals, patients, patient organisations and members of the public. The network should in this case include members with different backgrounds.

#### Conditions for involvement

Patients involved in the dissemination process have specific needs that should be taken into account. The GIN toolkit suggests (1) informing patients about their role before participating, (2) clarifying expectations about the specific role of the patients and the time commitment required, (3) giving a training in advance to prepare patients for their assigned role and (4) supporting patients during the process [38]. The training could be in technical areas such as how to understand the terminology or how to take part in the group effectively (e.g. assertiveness) [38]. Supporting patients can be done by providing networking opportunities for individuals or by providing a buddy [38].

#### Suggestions to make patient involvement more successful

Three suggestions aimed at creating patient involvement in the development and dissemination of recommendations are described in the literature. The first suggestion is the use of selection criteria in choosing patient representatives [22]. A criterion may be the ability to consider the evidence objectively and to make recommendations that do not depart from preconceived views or self-interests [22]. Second, involve patients in less traditional ways (e.g. as committee member) and choose for more innovative ways such as the use of new media that better fit the patients’ role, expectations and capabilities. The development group may not include a consumer representative but may invite patients to review draft documents or attend a group meeting or internet forum to share their perspectives [22]. The third suggestion is training: provide patients with sufficient information and knowledge before and during the project. This empowers them to become effective partners in the dissemination and implementation process [38].

## Discussion

This systematic review has identified 21 studies that describe how recommendations can be disseminated to patients and patient organisations. Such a review was missing and adds up to our knowledge on patient involvement in the development [2, 6] and dissemination of recommendations to patients and patient organisations and indirectly to professionals [7, 41].

At the start, we assumed a difference between dissemination towards professionals in comparison to dissemination to patients. In this discussion, we evaluate this assumption in the light of our results and existing literature.

This review shows that dissemination towards patients does not differ when it comes to the need to make a plan before or at the start of the project. It should take the needs and preferences of the target audience into account as well as contextual factors [7]. However, dissemination strategies to patients versus health professionals have to be different when it comes to the development process of recommendations, the language used and the methods and tools that are used for dissemination. First, recommendations should not be developed without the active involvement of patients.

Second, the original recommendations, often developed by and for professionals, need translation into a readable lay version for patients [6]. Not only are the scientific journals in which recommendations are published inaccessible for patients, they are also written in a language that cannot be understood by patients with low or moderate health literacy. The translation into a readable, comprehensible, relevant, consistent, unambiguous and credible version [6, 7, 16, 23–25] for patients will increase the success of the dissemination.

In some cases, patient friendly decision aids may also be an adequate tool for dissemination of recommendations to patients although more research is needed to define condition where decision aids can replace or complement lay versions of recommendations.

This review suggests that patients can be involved in making this lay version [1]. The selection of patients may be challenging. Patients are expected to be able to review scientific evidence objectively and to approach the recommendations from a wider patient perspective rather than individual preconceived views or self-interests [22]. Ideally, a heterogeneous group of patients with different educational levels [38] should be involved in order to communicate to a diversity of patients [22, 23, 37]. In several rheumatology patient networks, we have recognised that white, female and higher educated patients are often overrepresented [42]. A concern is that such misrepresentation of patients might lead to lay versions that are not serving the wider groups of patients something that is confirmed by the study from South Africa [25]. It is therefore important to involve a diverse group of patients (age, gender, educational level, ethnicity), and for that aim, the adaptation of existing processes might be needed to make their participation possible. A final concern may be the question whether recommendations are still valid when being translated or adapted. A solution as used by EULAR is the check and formal approval of the adapted English version by the task force leader and the chair of the standing committee of patients.

Third, dissemination strategies need to be tailored to the needs of patients that use other channels for communication and education than professionals. Although the findings of our study regarding the value of a multifaceted and active strategies are much in line with studies on dissemination towards professionals [43, 44], the applied tools and methods are different. Similar to the condition of patient involvement in the development of recommendations, patients and patient organisations should be actively encouraged to support and contribute to the actual dissemination.

Not only for ethical reasons but also because direct involvement improves the dissemination [1, 22–25, 36–38]; patients should be involved in the whole process. In particular, patient organisations can play an important role in reaching out to the patient community and to solve difficulties to reach patients. They can provide information on their websites, can coordinate web-communities, can organise self-management trainings and other educational events and publish patient magazines, books and brochures. Larger organisations run national campaigns and are able to generate funds to promote education on the disease management of their members. Strategies where health professionals and patient organisations join forces will probably be even better. In these cases, information about the recommendations may reach the patient from different sources with the benefit of mutual enforcement.

Although patients are more and more involved in the development of guidelines [3, 45] (for professionals), their involvement in the dissemination process (towards patients and professionals) is still less common. This may be a result of the problems professionals expressed on patient involvement in the development of recommendations [2]. Professionals reported the discrepancy between the perspectives of themselves and patients as an important barrier in the development of recommendations. They find it difficult to reconcile the preferences of patients with their own views [2]. At the same time, patients find it difficult to affirm their views and experience in the presence of evidence-based information and existing power asymmetries wherein expert knowledge is appreciated more than experiential knowledge [46–49]. Their lack of familiarity with the scientific and medical terminology made communication also more difficult [2]. By persisting the use of such language, professionals unintentionally exclude patients [50].

The involvement of patients is thus seen as important and as a key to success, but at the same time, it seems to be difficult to involve patients in an adequate way. Training and support for patients are described as helpful and are therefore needed to make the involvement of patients in the development and dissemination of recommendations successful [2, 22, 38] which in the end will lead to more successful dissemination of the guidelines to patients and patient organisations. Training for professionals should however also get attention since professionals also have to learn how to work effectively together with patients [51].

### Strengths and limitations

The strength of this systematic review is the fact that it for the first time focuses solely on the target group of patients and patient organisations, a target group previously often ignored. A limitation might be the low level of evidence of most included articles. This has been the result of our inclusion criteria. Because we expected to find a small number of relevant articles, we chose to be inclusive regarding their methodological quality. For this reason, the results of this review should not only be handled with care, it should also encourage researchers to initiate evaluation studies that will provide knowledge about the effectiveness of dissemination strategies with higher levels of evidence.

## Conclusions

Our study is the first systematic literature review that provides an overview of strategies to disseminate recommendations to patients or patient organisations and how to involve patients in this process. The findings were helpful to develop a practical guide for national patient organisations to promote the dissemination of recommendations among patients. The dissemination of recommendations towards patients should largely follow the same principles as dissemination towards professionals, although best practices are still scarce and procedures are not always followed. More attention should however be given to making a lay version which takes the diversity of the target group, patients, into account. Involvement of patients, not only in the dissemination of recommendation but also in the development of recommendations, is needed. The use of PAR and training for patients and professionals may be helpful to improve the quality of the participation of patients.

This review also identified a significant knowledge gap regarding effective dissemination strategies: More valid and credible research has to be conducted in order to obtain higher levels of evidence for the effects, efficiency and barriers of existing dissemination strategies and the role of patient organisations in that process.

## Additional files

Additional file 1: Search terms. (DOCX 22 kb)

Additional file 2: (PDF 57 kb)

Additional file 3: Quality Assessment Tool for Qualitative Studies. (DOC 47 kb)
