# Supplementary material for: Strategies for disseminating recommendations or guidelines to patients: a systematic review
Source: Implement Sci. 2016 Jun 7;11:82. doi: 10.1186/s13012-016-0447-x (PMC4895829; doi:10.1186/s13012-016-0447-x)
Supplement: Supplementary file 1 — Search terms. (DOCX 22 kb) [file 13012_2016_447_MOESM1_ESM.docx]

*Additional file 1: Search terms*

*Search strategy for PubMed (4 February 2016)*

[Mesh] = Medical subject headings, keywords

[tiab] = words in title or abstract

| **Search** | **Query** | **Items found** |
| --- | --- | --- |
| **#4** | #1 AND #2 AND #3 | **2,375** |
| **#3** | "Information Dissemination"[Mesh] OR disseminati*[tiab] | **53,767** |
| **#2** | "Guidelines as Topic"[Mesh] OR "Guideline" [Publication Type] OR guideline*[tiab] OR recommendation*[tiab] OR cpg[tiab] | **465,944** |
| **#1** | "Patients"[Mesh] OR "Consumer Organizations"[Mesh] OR patient[tiab] OR patients[tiab] OR client*[tiab] OR consumer*[tiab] OR people[tiab] OR public involvement*[tiab] OR ppip[tiab] OR "Consumer Participation"[Mesh] | **5,404,470** |

*Search strategy for Embase.com (4 February 2016)*

/exp = EMtree keyword with explosion

/de = EMtree keyword without explosion

:ab,ti = words in title or abstract

NEAR/x = words near to each other, x places apart

| **Search** | **Query** | **Items found** |
| --- | --- | --- |
| **#4** | #1 AND #2 AND #3 | **3,507** |
| **#3** | 'information dissemination'/exp OR disseminati*:ab,ti | **66,565** |
| **#2** | 'practice guideline'/de OR guideline*:ab,ti OR recommendation*:ab,ti OR cpg:ab,ti | **679,724** |
| **#1** | 'patient'/exp OR 'consumer'/exp OR patient:ab,ti OR patients:ab,ti OR client*:ab,ti OR consumer*:ab,ti OR people:ab,ti OR (public NEAR/3 involvement*):ab,ti OR ppip:ab,ti | **7,423,686** |

*Search strategy for Ebsco/PsycInfo (4 February 2016)*

DE = keyword

TI = words in title

AB = words in abstract

| **Search** | **Query** | **Items found** |
| --- | --- | --- |
| **S4** | S1 AND S2 AND S3 | **463** |
| **S3** | ( DE "Information Dissemination" OR DE "Knowledge Transfer" ) OR TI disseminati* OR AB disseminati* | **11,953** |
| **S2** | DE "Treatment Guidelines" OR TI ( guideline* OR recommendation* OR cpg ) OR AB ( guideline* OR recommendation* OR cpg ) | **116,124** |
| **S1** | ( DE "Patients" OR DE "Geriatric Patients" OR DE "Hospitalized Patients" OR DE "Medical Patients" OR DE "Outpatients" OR DE "Psychiatric Patients" OR DE "Surgical Patients" OR DE "Terminally Ill Patients" OR DE "Client Participation" ) OR TI ( patient OR patients OR client* OR consumer* OR people OR (public AND involvement*) OR ppip ) OR AB ( patient OR patients OR client* OR consumer* OR people OR (public AND involvement*) OR ppip ) | **903,303** |

*Search strategy for Ebsco/Cinahl (4 February 2016)*

MH = keywords; + = with explosion

TI = words in title

AB = words in abstract

| **Search** | **Query** | **Items found** |
| --- | --- | --- |
| **S4** | S1 AND S2 AND S3 | **497** |
| **S3** | (MH "Selective Dissemination of Information") OR TI disseminati* OR AB disseminati* | **5,075** |
| **S2** | (MH "Practice Guidelines") OR TI ( guideline* OR recommendation* OR cpg ) OR AB ( guideline* OR recommendation* OR cpg ) | **114,234** |
| **S1** | ( (MH "Patients+") OR (MH "Consumer Organizations+") ) OR TI ( patient OR patients OR client* OR consumer* OR people OR "public involvement" OR "public involvements" OR ppip ) OR AB ( patient OR patients OR client* OR consumer* OR people OR "public involvement" OR "public involvements" OR ppip ) | **820,195** |
