# Supplementary material for: Strategies for disseminating recommendations or guidelines to patients: a systematic review
Source: Implement Sci. 2016 Jun 7;11:82. doi: 10.1186/s13012-016-0447-x (PMC4895829; doi:10.1186/s13012-016-0447-x)
Supplement: Supplementary file 3 — Quality Assessment Tool for Qualitative Studies. (DOC 47 kb) [file 13012_2016_447_MOESM3_ESM.doc]

*Additional file 3: Quality Assessment Tool for Qualitative Studies*
